# Responsiveness and clinical utility of PROMIS instruments in pediatric Crohn's disease: insights from a longitudinal study

**DOI:** 10.3389/fped.2024.1473286

**Published:** 2025-01-15

**Authors:** Sara Azevedo, Maria Miguel Oliveira, Paulo Nogueira, Ana Isabel Lopes

**Affiliations:** ^1^ Gastroenterology Unit, Pediatrics Department, Santa Maria University Hospital—CHLN, Academic Medical Centre of Lisbon, Lisbon, Portugal; ^2^ Pediatrics University Clinic, Medical School, University of Lisbon, Lisbon, Portugal; ^3^ Área Disciplinar Autónoma de Bioestatística-Laboratório de Biomatemática, Medical School, University of Lisbon, Lisbon, Portugal; ^4^ Laboratório Associado TERRA, Instituto de Saúde Ambiental, Medical School, University of Lisbon, Lisbon, Portugal; ^5^ Centro de Investigação, Inovação e Desenvolvimento em Enfermagem de Lisboa, Escola Superior de Enfermagem de Lisboa, Lisbon, Portugal

**Keywords:** health-related quality of life, inflammatory bowel disease, pediatric Crohn's disease, patient-reported outcome measures, PROMIS, longitudinal study

## Abstract

**Background:**

Inflammatory bowel disease (IBD) may adversely affect physical, psychological, and social well-being. Integrating patient-reported outcomes (PROs) into clinical practice is crucial for comprehensive disease management.

**Objective:**

To evaluate the responsiveness and clinical utility of Patient-Reported Outcomes Measurement Information System (PROMIS) instruments, compared with standard clinical assessment tools in pediatric CD patients.

**Methods:**

A longitudinal, prospective study with 31 pediatric Crohn's disease (CD) patients aged 8–17 years recruited from a Pediatric Gastroenterology Unit. Data were collected at baseline and every 6 months over 18 months. PROMIS pediatric measures assessed PROs. Disease activity was evaluated using the pediatric Crohn's disease activity index (PCDAI) and clinical markers. IMPACT-III was also applied. Linear mixed-effects models (LMMs) and bivariate analyses were used to assess changes over time.

**Results:**

PROMIS Global Health scores showed significant improvement over time, indicating enhanced overall health perceptions among patients. Notable reductions were observed in PROMIS Pain Interference and Fatigue scores, indicating better physical health. PROMIS depression scores generally decreased, suggesting improved mental health. PCDAI scores, hemoglobin, and platelet count significantly changed and correlated with PROMIS measures. Globally, the study demonstrated significant and clinically relevant changes in multiple PROMIS measures, confirming their responsiveness to changes in disease activity.

**Conclusion:**

PROMIS instruments are clinically useful in managing pediatric CD, providing valuable insights into global health and quality of life. Integrating PROMIS measures into routine clinical practice may enhance disease management and treatment strategies for pediatric IBD patients.

## Highlights

• **What is already known?**

PROMIS pediatric measures are clinically meaningful and responsive over time.

• **What is new here?**

Assessing utility and responsiveness in a real clinical context by comparing standard clinical assessment tools in pediatric CD patients with 10 PROMIS pediatric measures

• **How can this study help patient care?**

PROMIS is valuable in pediatric CD management, providing insights into the patient's perspective, which is essential for a holistic understanding of the disease and its impact on daily life. Incorporating these measures can lead to more personalized and effective treatment strategies, ultimately enhancing patient-centered care.

## Introduction

Inflammatory bowel disease (IBD) is a chronic immune-mediated disease with worldwide increasing incidence and prevalence across both adult and pediatric populations (1, 2). In Europe alone, it is estimated that around 2 million individuals are affected by this condition (3), with 10%–25% of this diagnosis occurring during childhood and adolescence (4). Moreover, there has been a notable rise in IBD cases among very young children, particularly those under 6 years of age, classified as very early onset IBD (2, 5, 6). This increase underscores the significant disease burden and long-term morbidity that pediatric patients endure, impacting their health throughout their lives (7).

Pediatric IBD presents a unique psychological and emotional challenge for both patients and their caregivers. These families must manage a chronic disease characterized by an unpredictable course and relapsing symptoms, requiring lifelong immune-modify therapy treatments and frequent disease monitoring (8). Healthcare teams face the challenge of achieving stringent treatment goals (8, 9), such as inducing and maintaining deep remission (7), while also promoting normal growth, good emotional and psychological health, and minimizing the negative impacts of treatment and intensive monitoring (2, 8–11).

In recent years, there has been growing awareness of the importance of routinely integrating the patient perspective and satisfaction into clinical practice. Patient-reported outcomes (PROs), which are measures reported directly by the patient regarding the outcome of treatments and disease management, have become critical in this context (12, 13). These measures offer valuable insights into patient's experience with the disease and its treatment, highlighting aspects of disease management that might be overlooked by traditional clinical assessments (13). In pediatric settings, PROs are increasingly used as important endpoints in both comparative effectiveness research and clinical practice for various chronic conditions (14–16). Moreover, PROs serve as an exemplary model for capturing the perspectives of both patients and parents in the context of pediatric CD. This approach facilitates a more comprehensive integration of the pediatric patient perspective within the disease management process. Healthcare providers are encouraged to prioritize disease management and decision-making in pediatric patients while recognizing the pivotal role of parents in the successful management of the disease.

The Patient-Reported Outcome Measurement Information System (PROMIS) is a comprehensive system of PRO designed to assess various domains of health, including physical, psychological, and social health, as well as health-related quality of life (HRQOL) (https://www.nihpromis.org). PROMIS pediatric measures specifically validated for children aged 9–17 years have demonstrated validity, reliability, and responsiveness across several chronic conditions (15–18), including pediatric (19, 20) and adult (21) IBD. Understanding the impact of medical interventions on the well-being and HRQOL) among IBD patients is critical, especially as treatment goals become more precise and new treatments become available. When managing IBD patients, a comprehensive approach to function, disability, and general well-being, in addition to traditional clinical parameters, is desirable (22). There is consensus on the importance of a global and comprehensive approach to the care of pediatric IBD patients, including their perspective of the disease, treatments, and follow-up (22). Reliable and validated PRO measures that are responsive to changes in disease activity are essential for effective disease management (19, 20, 23, 24) in clinical practice. However, the clinical usefulness of PROs depends on their ability to reflect changes in the disease over time, as indicated by disease markers (17, 25). The concept of responsiveness to change refers to the ability of a scale to detect and quantify changes in a patient's condition over time in a meaningful way. This is crucial for both patients and clinicians to assess modifications in disease activity accurately (24). Minimally important differences (MIDs) represent the smallest changes in PRO scores that can indicate a clinically meaningful change in the outcome being measured. Previous studies suggest MIDs of 2–3 for multiple pediatric PROMIS instruments (17, 18, 24, 26).

The present study aims to evaluate the responsiveness of PROMIS over time (10 short-form pediatric PROMIS measures), in comparison with traditional medical assessments, in a cohort of pediatric CD patients in a real clinical setting.

## Materials and methods

### Study design and patient selection

**Figure 1:**
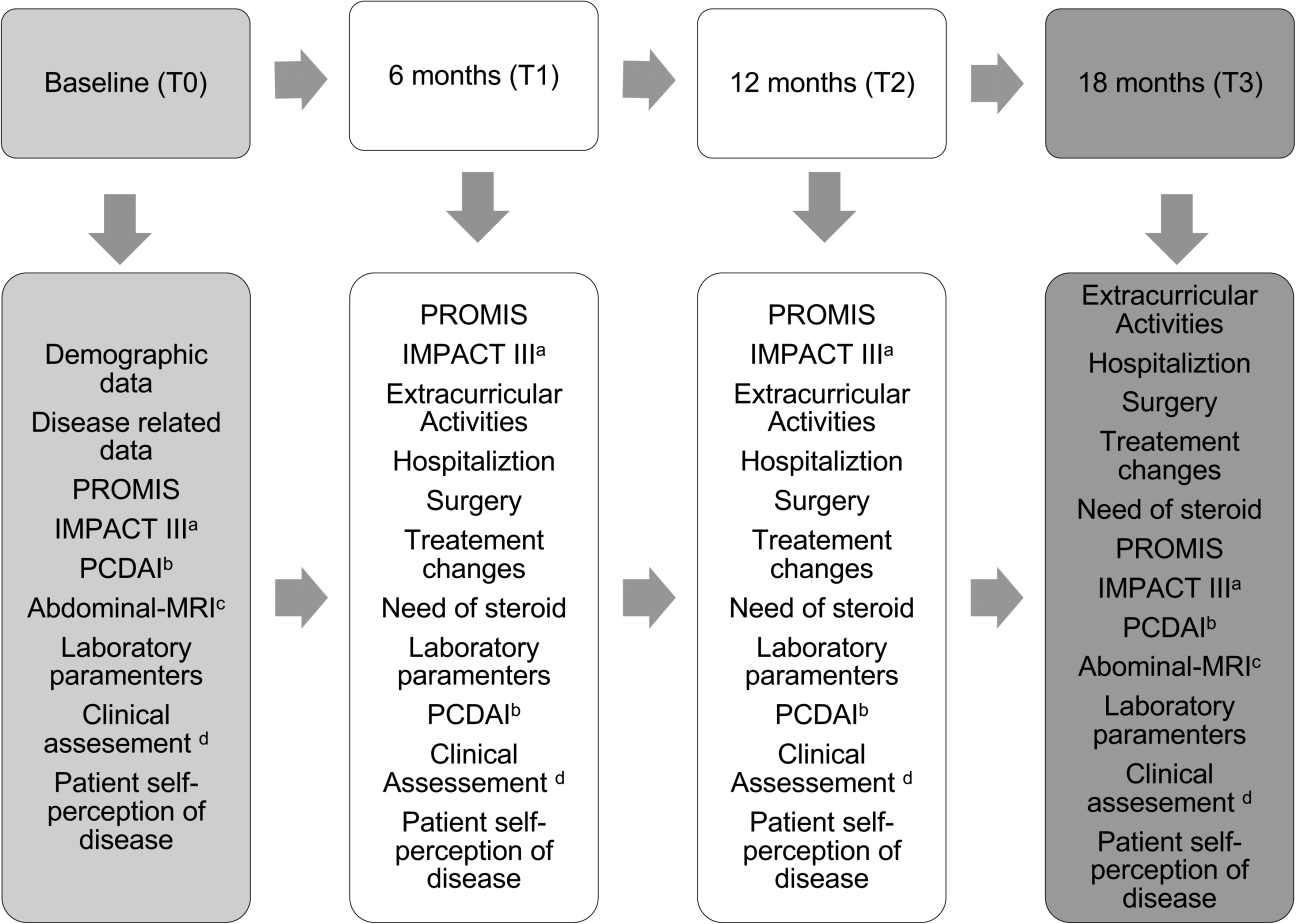
Study design. (a) IMPACT III 35-item self-administered questionnaire of health-related quality of life in pediatric IBD, score ranges 35 (poor) to 175 (best); (b) pediatric Crohn’s disease activity index-PCDAI scoring < 10 were considered in remission; (c) abdominal-MRI = abdominal-magnetic resonance imaging: (d) please refer to Figure 2.

This is a longitudinal, prospective study involving all pediatric CD patients aged 9–17 years and 364 days, recruited from a single Pediatric Gastroenterology Unit Reference Center in an outpatient setting (convenience sample). The follow-up period spanned 18 months, with data collection carried out between 2 January 2021, and 28 February 2023. Participants completed surveys at baseline and 6-month intervals during their scheduled appointment (Figure 1). Patients were stratified into two groups based on pediatric Crohn's disease activity index (PCDAI)—PCDAI (24, 25): remission group PCDAI <10 (G1) and active disease group PCDAI >10 (G2) at each survey interval. Informed assent (for patients under 16 years) and consent (for patients over 16 years and caregivers of patients under 16 years) were obtained before enrollment. Patients who did not sign the written assent/consent, those with limitations in verbal or written comprehension of Portuguese, hospitalized patients, and recently diagnosed patients (<1 month) were excluded.

#### Sample size and power

This study used a convenience sample based on the total number of eligible patients followed at the unit during the recruitment period, adhering to the inclusion and exclusion criteria. The final sample comprised 31 participants. While efforts were made to maximize the sample size, the limited pool of patients within the study timeline was a constraint. Post hoc power analysis indicates that with 31 patients, the study achieved approximately 57.8% power to detect mean differences of 0.8 units in PROMIS measures with a standard deviation of 2.0. This power limitation is acknowledged as a constraint in detecting smaller effect sizes.

### Data collection

**Figure 2:**
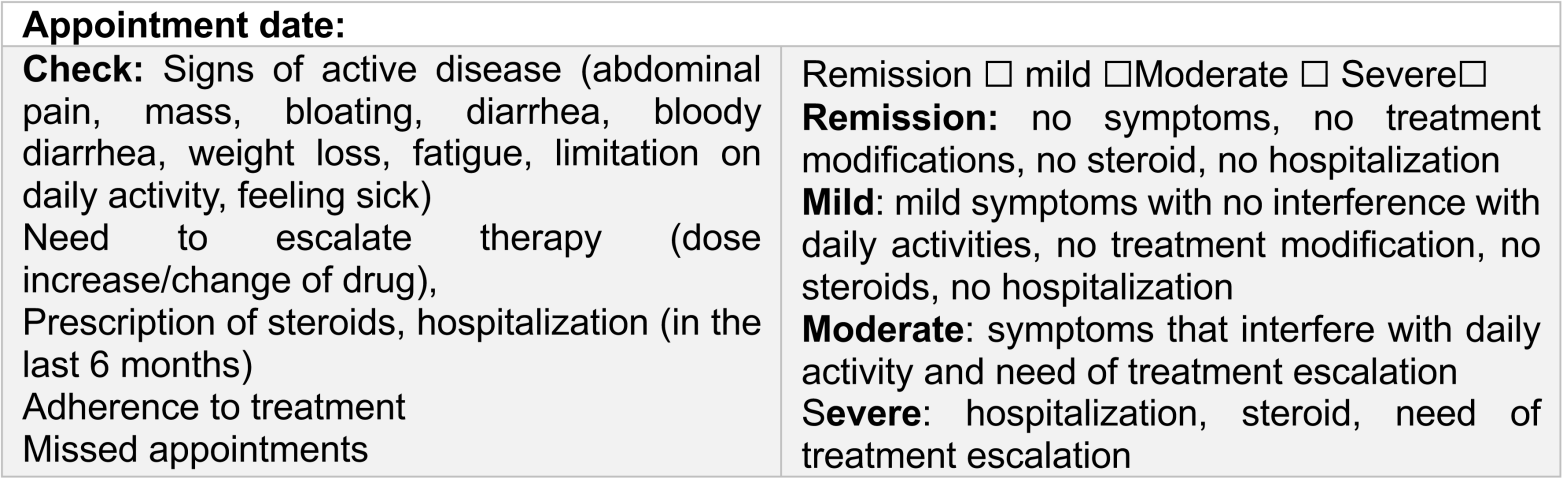
Clinical physician global assessment of disease.

All data were collected by the same investigator who also performed the clinical assessment (Figure 2) at baseline and during the scheduled appointments (every 6 months). All pediatric patients completed the questionnaires in the waiting room before the medical appointment or during treatment infusions. Clinical and demographic data were collected from patients' records and supplemented with a brief questionnaire covering academic data and extracurricular/social activities. Different types of data were collected during the study assessment periods (Figure 1) and included the following.

#### Demographic data

Gender, birth date, age at diagnosis, school level, extracurricular activities. The levels of education were classified according to the International Standard Classification of Education (ISCED 2011) [Commission Regulation (EU) No 317/2013]. ISCED 2011 has nine education levels, from Level 0 to Level 8.

#### Disease-related data

Time of diagnosis, disease duration in years, disease phenotype (Paris Classification) (27), need for hospitalization and/or surgery at enrollment, treatment at enrollment, and need for treatment escalation (in the prior 6 months and during follow-up).

#### Anchors’ assessments of disease

Disease activity was assessed using the PCDAI (28, 29), with scores <10 considered indicative of remission. Complementary exams included: abdominal magnetic resonance imaging (MRI) and laboratory tests: hemoglobin (Hb), hematocrit, platelet count, erythrocyte sedimentation rate (ESR), C-reactive protein (C-RP), serum albumin, serum ferritin, and fecal calprotectin (FC) levels. Clinical disease severity was assessed by the physician and categorized as remission, mild, moderate, or severe (Figure 2).

#### Patient self-perception of disease status

A simple semiquantitative scale was applied to patients with regard to the past 6 months (interappointment interval). Patients rated their disease status on a semiquantitative scale: “feeling better/good, feeling the same/not good, not bad, feeling bad/worsening.”

The subjective patient/caregiver self-perception of disease control (30) was based on the patient's notion of disease control (17, 31), including its perception of the global absence of symptoms or minimal symptoms, sense of social and emotional wellbeing, absence of pain or good pain control, and notion of treatment adequacy and or efficacy (30, 31).

#### Measure of health-related quality of life

To assess HRQOL, the IMPACT-III questionnaire was used (32). IMPACT-III is a 35-item self-report, IBD-specific measure of HRQOL, assessing six domains (bowel symptoms, systemic symptoms, social/functional concerns, body image, test and treatment concerns, and emotional concerns) using a 5-point Likert scale. The score ranges from 35 (poor) to 175 (best), with lower scores indicating poorer HRQOL.

#### PROMIS measures

Ten short-form pediatric PROMIS measures, with a fixed set of 4–10 items, were selected to access PROs: global health, meaning and purpose, cognitive function, life satisfaction, peer relationship, depression, anxiety, pain interference, physical activity, and fatigue. PROMIS instruments were calibrated using a T-score metric with the mean of the original calibration population equal to 50 and the standard deviation (SD) in the calibration population equal to 10 (33). Higher PROMIS pediatric scores indicated more of the measured domain (16). This study considered MIDs significant if ≥3 for multiple pediatric PROMIS instruments (26).

### Statistical analysis

Descriptive statistics were used to summarize the demographic and clinical characteristics of the participants. Paired sample *t*-tests were used to assess differences between PROMIS measures at individual time points, while the Friedman test was applied to evaluate changes in clinical assessments and disease self-perception over time. Longitudinal changes in PROMIS measures were evaluated using linear mixed models (LMMs), which accounted for repeated measures and included fixed effects for time, sex, age, educational level, and treatment type, with random intercepts for each subject. LMM was selected to account for the nested structure of repeated measures within participants, enabling the analysis of temporal changes while controlling for individual variability.

This study was designed to explore temporal trends in PROMIS measures rather than conduct sensitivity analyses or directly assess associations with disease activity. This reflects the observational nature of the study and the constraints of the available sample size. While no direct statistical tests of association between PROMIS measures and disease activity markers were performed, model coefficients were compared to assess common directional trends. The minimally important difference (MID) threshold of 3 was used to determine clinically significant changes in PROMIS scores.

## Results

### Study population

The study included 31 participants, of whom 58% were female (*n* = 18), with a mean age of 15.3 (±2.0). Three patients were lost to follow-up during the study. The mean age at CD diagnosis was 12.7 (±3.4) years, and the mean disease duration was 2.7 years (±2.7). Complete demographic and clinical data are presented in Table 1.

**Table 1:** Demographic and disease-related data at baseline.

Gender M/F (%) 13/18 (41.9/58.1)	
Age, years, mean (SD)	15.2 (±2)
Level of education (ISCED)^a^ *n* (%)	ISCED 2: 6 (19.4) ISCED 3–5: 25 (80.6)
Extracurricular activities *n* (%)	9 (29.0%)
Disease duration, years in T, mean (SD)	2.7 (±2.7)
Age at diagnosis years, mean (SD)	12.7 (±3.4)
Paris classification, age of onset *n* (%) A1a 7 (22.6%)/A1b 24 (77.4%)	
Paris classification, location *n* (%) L1 (distal 1/3 ileal + limited cecal disease): 1 (3.2%) L2 (colonic): 3 8 (9.6%) L3 (ileocolonic): 18 (58.1%) L3L4a (ileocolonic + upper discase proximal to the Ligament of Treitz):8 (25.9%) L2L4a (colonic + upper discase proximal to the Ligament of Treitz): 1 (3.2%)	
Paris vphenotype *n* (%) B1 (non-stricturing non-penetrating): 27 (87.1%) B2 (stricturing): 3 (9.7%) B3 (penetrating): 1 (3.3%) Perianal disease 5 (16.1%)	
Paris growth *n* (%) G0 (no evidence of growth delay): 27 (87.1%) G1 (growth delay): 4 (12.9)	
Treatment at baseline *n* (%)	Immunomodulator 15 (48.4%) Immunomodulator + EN^b^ 1 (3.2%) Immunomodulator + PDN^c^ 2 (6.5%) Anti TNF alfa^d^ 13 (41.9%)
Number of biologics until baseline *n*/31 (%)	11/31 (35.5%) 1 biologic 3/11 (9.7%) 2 biologics
Need for hospitalization *n* (%) ^e^	3 (9.7%)
Need for surgery *n* (%) ^e^	0 (0%)
Treatment modifications *n* (%) ^e^	3 (9.7%
Need for corticosteroids *n* (%) ^e^	2 (6.5%)
Poor compliance to treatment *n* (%) ^e^	3 (9.7%)

^a^ ISCED (International Standard Classification of Education): ISCED 0–2: Lower secondary education, ISCED 3–5: Upper secondary education and ISCED > 6 Higher education.

^b^ EN, enteral nutrition.

^c^ PDN, prednisolone.

^d^ Anti-TNF alfa, antitumoral necrosis factor alfa.

^e^ Prior 6 months before baseline.

SD, standard deviation.

At baseline, 70.9% (*n* = 22) of participants were considered to be in remission according to the physician assessment (Table 2), with a mean PCDAI of 7.5 (±9.8). With regard to biochemical remission, although serological markers were within normal ([mean Hb o13 (±1.3) g/dL, CRP 0.6 (±1.7) mg/dL, ESR 21 (±19.8), and Ferritin (±1,189.7) µg/dL)], the mean FC of 803.7 (±1,189.7) µg/g indicated active disease, with 32% (*n* = 10) presenting FC < 250 µg/g. Imaging revealed inactive disease in 41.9% (*n* = 13) of participants.

**Table 2:** Anchor assessment of disease, HRQOL, and PROMIS scores over time.

	T0 (*n* = 31)	T1 (*n* = 31)	T2 (*n* = 31)	T3 (*n* = 28)
Abdominal MRI^a^ *n* (%)				
Absence of activity (remission)	13 (41.9)	–	–	13 (46.4)
Persistent active disease inflammation	16 (51.6)	–	–	14 (50)
Stricturing and fistulizing disease:	1 (3.2)	–	–	1 (3.6)
Laboratory test mean (SD)				
Hb^b^ (g/dL)	13 (±1.3)	13.1 (±1.2)	13.0 (±1.5)	13.4 (±1.2)
Hematocrit (%)	38.4 (±3.9)	38.9 (±3.6)	38.6 (±4.2)	40 (±3.3)
Platelets (109/L)	330.3 (±81.0)	291.5 (±69.5)	279.2 (±56.2)	255.1 (±47.0)
ESR^c^ (mm)	21 (±19.8)	25.5 (±23.2)	21.6 (±18.9)	16.7 (±13.4)
CRP^d^ (mg/dL)	0.6 (±1.7)	0.7 (±1.3)	0.7 (±1.3)	0.4 ± (0.9)
Ferritin (µg/dL)	81.2 (123.8)	60.3 (±51.5)	49.8 (±37.0)	81.3 (±114.7)
Fecal calprotectin (µg/g) Fecal calprotectin < 250 µg/g Patients *n* (%)	803.7 (±1,189.7) 10 (32.3)	846.2 (±1,364.8) 14 (45.2)	533.0 (±607.7) 11 (35.5)	427.5 (±110.0) 15 (51.7)
PCDAI mean (SD)^e^	7.5 (±16.1)	4,9 (±6.2)	5.8 (±2.5)	3.3 (±2.5)
PCDAI *n* (%) inactive	25 (80.6)	29 (93.5)	25 (80.6)	28 (96.5)
PCDAI *n* (%) mild-to-moderate	6 (19.4)	2	6 (10.4)	1 (3.5)
PCDAI *n* (%) moderate-to-severe	0	0	0	0
Physician clinical assessment score *n* (%)				
Remission	22 (70.9)	23 (74.2)	22 (71)	21 (75)
Mild	6 (19.4)	4 (12.9)	9 (29)	7 (25)
Moderate	4 (12.9)	4 (12.9)	0	0
Severe	0	0	0	0
Indicators of poor outcome during follow-up *n* (%)				
Hospitalization^f^	0 (0)	0 (0)	0 (0)	0 (0)
Surgery^f^	0 (0)	0 (0)	0 (0)	0 (0)
No compliance to treatment^f^	3 (9.7)	1 (3.2)	1 (3.2)	6.9 (2)
Treatment with steroid^f^	2 (6.4)	1 (3.2)	1 (3.2)	0 (0)
Treatment escalation	3 (9.7)	8 (25.8)	5 (16.1)	4 (13.8)
Patient self-perception of disease *n* (%)				
Feeling better	11 (35.5)	12 (38.7)	6 (19.3)	7 (25)
Feeling worse	6 (19.3)	0	1 (3.3)	0
Feeling the same	14 45.2)	19 (61.3)	24 (77.4)	21 (75)
IMPACT III^g^ mean (SD)	73.2 (±13.1)	77.2 (±15.1)	78.2 (±15.1)	80.4 (±12.7)
PROMIS pediatric scores T-scores mean (SD)				
Global health	43.2 (±7.9)	43.6 (±9.1)	45.9 (±9.6)	46.8 (±8.6)
Depression	51.1 (±12.7)	47.1 (±13.4)	43.2 (±8.0)	45.4 (±7.8)
Anxiety	50.4 (±9.1)	46.6 (±10.4)	50.4 (±9.1)	46.6 (±10.5)
Meaning and purpose	40.1 (±8.1)	42.0 (±10)	41.6 (±9.2)	43.1 (±9.7)
Pain	47.3 (±12.6)	43.1 (±10.7)	43.5 (±11.5)	41.2 (±9.9)
Cognitive function	46.7 (±6.2)	47.5 (±7.0)	46.5 (±7.9)	46.9 (±8.4)
Life satisfaction	44.1 (±10.2)	47.4 (±9.5)	45.5 (±8.6)	46.2 (±8.0)
Peer relationship	50.6 (±9.5)	49.8 (±9.0)	50.5 (±11.3)	51.1 (±10.9)
Physical activity	43.0 (±7.2)	44.5 (±6.7)	46.6 (±7.3)	43.8 (±7.8)
Fatigue	52.4 (±12.1)	50.6 (±11.7)	48.7 (±11.2)	48.6 (±11)

^a^ Abdominal MRI, abdominal magnetic resonance imaging.

^b^ Hb, hemoglobin.

^c^ ESR, erythrocyte sedimentation rate.

^d^ CRP, C-reactive protein.

^e^ Pediatric Crohn's disease activity index-PCDAI scoring. Inactive disease: PCDAI 0–10; Mild-to-moderate disease: >10–32.5; Moderate-to-severe disease: >32.5.

^f^ Prior 6 months to enrollment.

^g^ IMPACT III 35-item self-administered questionnaire of health-related quality of life in pediatric IBD, score ranges 35 (poor) to 175 (best).

SD, standard deviation.

Most participants (80.6%, *n* = 25) perceived their health condition as “better or the same” compared with the previous 6 months, with a mean IMPACT-III score of 73.2 (±13.1). During follow-up, clinical stability was observed in most patients, with mean PCDAI scores remaining in the inactive range despite persistently elevated FC values. Treatment intensification with biological agents was required in 54.8% (*n* = 17) of participants, but none required hospitalization.

### PROMIS measures

**Figure 3:**
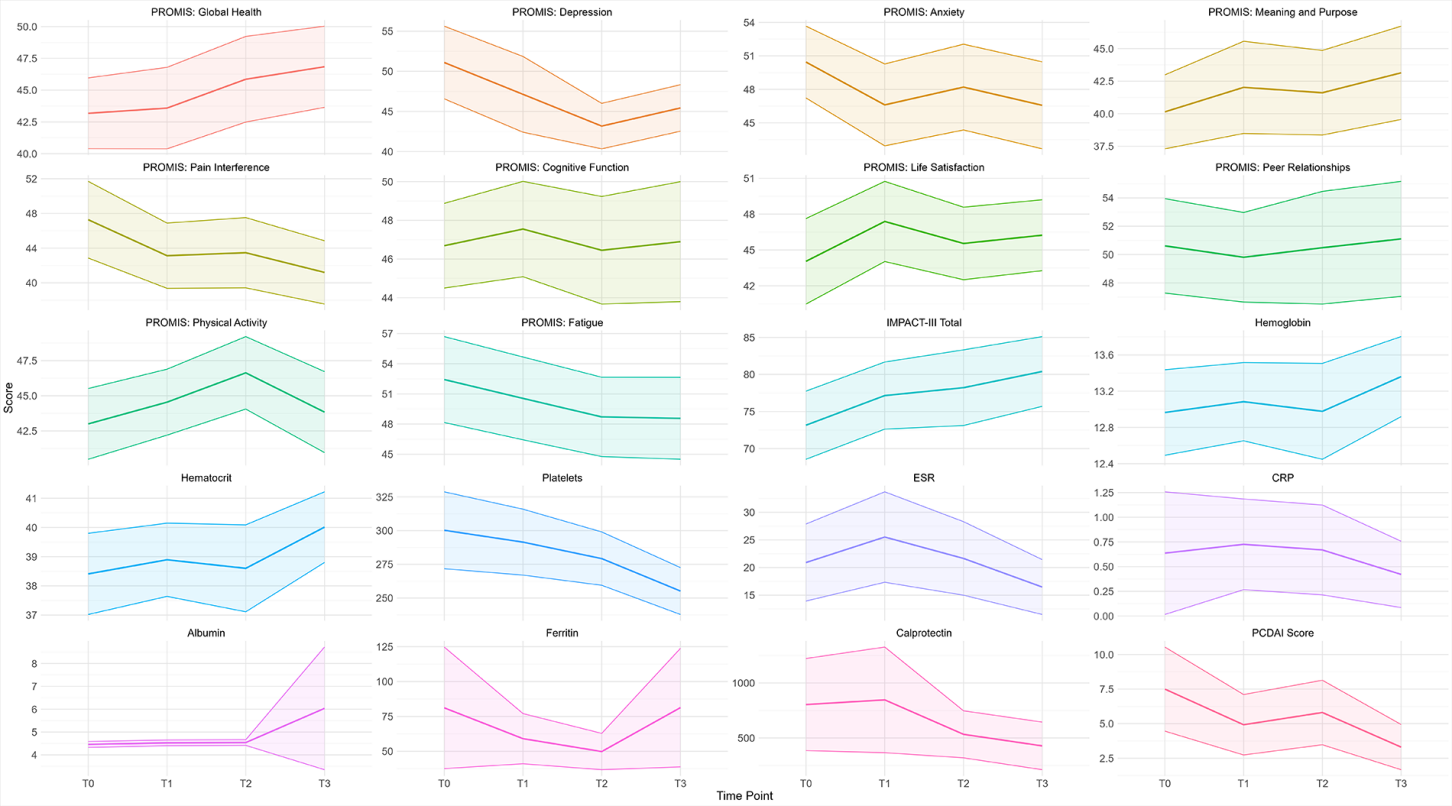
PROMIS measures, total IMPACT-III, and laboratory measures the mean trend throughout the study period. The shaded ribbons represent the 95% confidence interval.

PROMIS scores exhibited distinct temporal trends over the study period (Table 2, Figure 3). Global Health showed significant improvements at T2 and T3, while Pain Interference and Fatigue consistently decreased, stabilizing in later time points. Depression and Anxiety showed early improvement during the study period but fluctuated at T3. PROMIS Physical Activity peaked at T2, while Life Satisfaction peaked at T1, showing the highest reported levels early in the study.

A bivariate analysis revealed significant differences in pain interference scores between T0 and T1 (*p* = 0.040) and in physical activity scores between T2 and T3 (*p* = 0.030). LMM analyses identified statistically and clinically meaningful changes (exceeding the MID threshold of 3) in several PROMIS measures (Table 3):

• Global Health improved significantly at T2 (*β* = −3.230, *p* < 0.05) and T3 (*β* = −5.695, *p* < 0.01).
• Fatigue improved at T2 (*β* = −4.809, *p* < 0.05) and T3 (*β* = −7.664, *p* < 0.05).
• Pain Interference improved consistently across all time points (e.g., T1: *β* = −4.401, *p* < 0.05; T3: *β* = −6.343, *p* < 0.01).
• Depression, Anxiety, and Life Satisfaction showed similar improvement trends early in the study.

**Table 3:** Linear mixed model unstandardized coefficient results for all PROMIS measures and total IMPACT-III scores.

	PROMIS measures							IMPACT-III^a^			
	Global health	Depression	Anxiety	Meaning and purpose	Pain interference	Cognitive function	Life satisfaction	Peer relationships	Physical activity	Fatigue	
Follow-up 1 (T0-T1)	0.855 (1.323)	−3.853 (2.897)	−4.306* (1.818)	1.915 (1.461)	−4.401* (1.906)	1.154 (1.096)	3.648* (1.716)	−1.020 (1.878)	1.805 (1.392)	−2.146 (1.873)	4.167* (2.028)
Follow-up 2 (T1-T2)	3.230* (1.420)	−8.838** (2.918)	−3.414 (1.909)	1.299 (1.587)	−4.474* (2.022)	0.108 (1.183)	2.136 (1.793)	−0.355 (1.964)	3.454* (1.443)	−4.809* (2.001)	5.864** (2.200)
Follow-up 3 (T2-T3)	4.494** (1.738)	−6.167 (3.168)	−5.305* (2.263)	3.089 (1.969)	−6.343** (2.435)	1.215 (1.457)	3.515 (2.108)	−0.198 (2.313)	0.308 (1.675)	−5.695* (2.433)	6.870* (2.726)
Female	−7.691** (2.656)	1.291 (2.176)	7.477* (3.021)	−2.624 (3.166)	4.809 (3.489)	−5.984** (2.285)	−5.183 (2.701)	−5.579 (2.987)	−4.853* (2.005)	6.418 (3.628)	−9.912* (4.368)
Age	−0.580 (0.675)	0.635 (0.569)	1.212 (0.774)	−0.060 (0.802)	0.444 (0.890)	−0.489 (0.580)	−0.542 (0.693)	0.468 (0.766)	0.083 (0.516)	1.555 (0.924)	−0.519 (1.107)
Secondary school or University	−1.560 (1.764)	1.288 (2.307)	−1.091 (2.283)	0.760 (1.986)	2.420 (2.474)	0.169 (1.475)	−1.496 (2.111)	−1.739 (2.320)	−0.188 (1.648)	0.928 (2.473)	−1.269 (2.752)
Non-biological treatment	−1.019 (1.495)	−2.576 (2.169)	−1.097 (1.971)	−1.646 (1.672)	−2.252 (2.115)	−1.256 (1.247)	−1.073 (1.832)	−0.798 (2.011)	−1.288 (1.444)	1.336 (2.103)	−0.340 (2.319)
Constant	58.000*** (10.806)	41.288*** (9.160)	28.887* (12.367)	43.012*** (12.841)	37.468** (14.240)	58.210*** (9.280)	56.825*** (11.077)	48.229*** (12.246)	45.378*** (8.241)	23.692 (14.780)	87.789*** (17.720)

* *p* < 0.05.

** *p* < 0.01.

*** *p* < 0.001.

^a^ IMPACT III 35-item self-administered questionnaire of health-related quality of life in pediatric IBD, score ranges 35 (poor) to 175 (best).

These findings highlight temporal trends in PROMIS measures, consistent with the study's aim to explore their longitudinal changes.

### Responsiveness clinical and laboratory measures

No statistically significant changes were found in laboratory measures between individual time points (Table 4). However, hematocrit increased significantly at T3, and platelet count decreased significantly at T2 and T3. PCDAI scores showed a significant reduction by T3 (*p* < 0.05), indicating overall clinical improvement over the study period (Figure 3).

**Table 4:** Linear mixed model unstandardized coefficient results for laboratory measures.

	Laboratory measures								
	PCDAI^a^	Hemoglobin	Hematocrit	Platelet	ESR^b^	C-RP^c^	Albumin	Ferritin	Fecal calprotectin
Follow-up 1 (T0-T1)	−2.822 (1.473)	0.142 (0.197)	0.621 (0.504)	−8.502 (12.746)	4.002 (3.008)	0.064 (0.258)	0.061 (0.926)	−24.258 (21.347)	97.136 (220.894)
Follow-up 2 (T1-T2)	−1.763 (1.518)	0.113 (0.207)	0.415 (0.535)	−25.103 (13.315)	0.159 (3.284)	0.046 (0.270)	−0.009 (0.934)	−33.371 (21.753)	−238.580 (231.530)
Follow-up 3 (T2-T3)	−3.501* (1.743)	0.313 (0.245)	1.282* (0.643)	−42.515** (15.657)	−4.300 (4.104)	−0.142 (0.319)	1.584 (1.019)	−2.482 (24.393)	−214.644 (262.651)
Female	0.096 (1.951)	−1.743*** (0.328)	−5.200*** (0.918)	10.541 (20.079)	1.449 (6.759)	−0.388 (0.419)	−0.723 (0.739)	11.294 (23.019)	−231.879 (306.731)
Age	−0.109 (0.503)	−0.048 (0.084)	−0.069 (0.234)	1.055 (5.153)	0.238 (1.708)	0.049 (0.107)	−0.0001 (0.193)	3.415 (5.970)	−90.146 (77.417)
Secondary school or University	0.781 (1.677)	−0.132 (0.247)	0.119 (0.654)	20.485 (15.680)	7.425 (4.119)	−0.441 (0.321)	0.656 (0.771)	−36.084 (21.707)	86.021 (252.878)
Non-biological therapeutics	−0.415 (1.483)	−0.143 (0.214)	0.048 (0.559)	4.695 (13.610)	5.380 (3.458)	0.291 (0.278)	−0.638 (0.721)	−12.411 (19.655)	−157.651 (235.156)
Constant	8.851 (8.041)	14.863*** (1.344)	42.379*** (3.749)	262.926** (82.338)	8.934 (27.366)	0.232 (1.718)	4.827 (3.103)	51.473 (95.451)	2,340.054 (1,233.208)

* *p* < 0.05; **p < 0.01; ***p < 0.001.

^a^ PCDAI, pediatric Crohn's disease activity index.

^b^ ESR, erythrocyte sedimentation rate.

^c^ CRP, C-reactive protein.

Some overlapping trends were observed between PROMIS scores and clinical measures. For example, pain interference scores appeared to decrease alongside reductions in PCDAI scores, and improvements in Global Health and Life Satisfaction coincided with increases in hematocrit. However, no direct statistical tests were performed to assess these associations. While these findings are exploratory, they suggest that PROMIS scores may capture aspects of patient health that complement traditional clinical markers.

### PROMIS trends and patient-reported outcomes

PROMIS measures, such as Global Health, Life Satisfaction, and Pain Interference, showed trends aligning with patient-reported outcomes, including IMPACT-III scores and self-perception of disease stability. Patients reporting stable or improved disease perception at baseline exhibited concurrent improvements in PROMIS scores over time. However, patients reporting worsening disease perception showed more variable PROMIS trends.

Both IMPACT-III scores and PROMIS measures demonstrated positive trends during the study period. Depression, Anxiety, Pain Interference, and Fatigue followed expected decreasing trends as patients reported improved wellbeing. These findings reflect an overall pattern of improved patient-reported outcomes but remain exploratory, as causal relationships cannot be established within this study.

### Physician clinical assessment and disease self-perception

Table 5 summarizes physician clinical assessments and patient self-perception of disease over time. No statistically significant differences were observed in physician clinical assessment scores between time points. However, multinomial regression models indicated that patients with a “Moderate” disease score were significantly less likely to achieve remission at T2 and T3 compared with T0 (*p* < 0.001). Patients with a “Mild” disease score showed no significant change in remission likelihood over time.

**Table 5:** Physician clinical assessment and patient self-perception of disease over time according to disease activity.^a^

		T0 (*n* = 31) G1 = 25 G2 = 6	T1 (*n* = 31) G1 = 29 G2 = 2	T2 (*n* = 31) G1 = 25 G2 = 6	T3 (*n* = 28) G1 = 25 G2 = 3
Clinical assessment score *n* (%)					
G1	Remission	18 (72.0)	22 (75.9)	17 (68.0)	18 (72.0)
	Mild	6 (24.0)	4 (13.7)	8 (32.0)	7 (28.0)
	Moderate	1 (4.0)	3 (10.3)	–	–
	Severe	–	–	–	–
G2	Remission	4 (66.7)	1 (50)	5 (83.3)	3 (100)
	Mild	–	–	1 (16.7)	–
	Moderate	2 (33.3)	1 (50)	–	–
	Severe	–	–	–	–
Patient self-perception of disease *n* (%)					
G1	Feeling better	8 (32.0)	10 (34.4)	4 (16.0)	6 (24.0)
	Feeling worse	3 (12.0)	–	1 (4.0)	–
	Feeling the same	14 (56.0)	19 (65.5)	20 (80.0)	19 (76.0)
G2	Feeling better	3 (50)	1 (50)	2 (66.7)	1 (33.3)
	Feeling worse	–	1 (50)	–	–
	Feeling the same	3 (50)	–	4 (33.3)	2 (66.7)

^a^ Disease activity determined according to the PCDAI score. A PCDAI score of >10 was considered an active disease.

Bivariate analysis of self-perception revealed significant differences between T1 and T2 (*p* = 0.01) and between T1 and T3 (*p* = 0.03). Patients perceiving their disease as stable (“feeling the same” or “better”) at baseline were more likely to report improvement at later time points. These trends align with broader improvements in PROMIS measures, although causal relationships were not tested.

## Discussion

This longitudinal study evaluated the responsiveness and clinical utility of PROMIS pediatric instruments in pediatric CD patients, comparing them with standard outcome assessment tools and recent international guidelines (34). The study followed participants over 18 months, during which PROMIS measures captured temporal trends in health status and quality of life (QOL), contributing to an improved understanding of patient-reported outcomes (PROs) in this population. These findings align with prior research suggesting the utility of PROMIS measures in capturing meaningful aspects of patient health, although further studies are necessary to establish their sensitivity and broader applicability.

### Summary of findings

PROMIS measures, particularly Global Health, Pain Interference, and Fatigue, showed significant improvements over the study period, reflecting better physical and emotional health among participants. These trends coincided with overall clinical stability, as indicated by PCDAI scores, platelet counts, and hematocrit levels. While trends in PROMIS measures, such as Pain Interference and Global Health, appeared to align with changes in the PCDAI and hematocrit, these findings are observational and require further statistical validation to confirm their significance. However, PROMIS measures also varied independently of objective clinical markers, such as fecal calprotectin (FC), emphasizing the unique insights that PROMIS instruments provide into patient-perceived health and QOL. This finding aligns with previous studies that underscore the independence of PROs from traditional clinical markers in pediatric IBD (9, 34).

Some PROMIS domains, including Depression and Anxiety, exhibited fluctuations rather than consistent trends, highlighting the complex interplay between physical symptoms, psychological wellbeing, and external factors and the multifaceted nature of pediatric IBD, underscoring the importance of integrating psychosocial dimensions into disease management (35–37). PROMIS measures, particularly those addressing emotional health, provide valuable insights into how physical symptoms interact with psychological and social wellbeing. These findings emphasize the complex nature of managing pediatric CD and the necessity of considering both physical and psychosocial dimensions of health in clinical practice.

### Comparison with previous studies

Our findings align with earlier longitudinal research, such as Arvanits et al., which suggested that PROMIS measures respond to changes in disease activity over time. More recently, two longitudinal studies using a web-based cohort of pediatric IBD patients (19, 20) evaluated PROMIS pediatric measures, including domains of pain interference, anxiety, depression, fatigue, and peer relationships. These studies demonstrated significant associations between PROMIS scores and disease activity, documenting clinically meaningful changes in PROs related to clinical markers and QOL. While our study similarly observed trends in PROMIS measures consistent with clinical improvement, the lack of direct statistical testing of these associations limits our ability to draw definitive conclusions.

Interestingly, we found limited changes in IMPACT-III scores compared with PROMIS measures. IMPACT-III, as a disease-specific HRQOL instrument, focuses primarily on IBD-related QOL, whereas PROMIS captures a broader range of physical, emotional, and social health domains. This distinction may explain the discrepancies observed and highlight the advantage of PROMIS instruments for holistic health assessment in pediatric CD patients.

### Influence of external factors

The timing of this study, coinciding with the COVID-19 pandemic, likely influenced PROMIS scores. At baseline, PROMIS measures reflected the negative impact of pandemic-related restrictions on physical and emotional wellbeing. As restrictions eased and access to healthcare improved, PROMIS scores also improved, particularly in domains such as Global Health and Life Satisfaction. Previous studies have documented the pandemic's impact on IBD patients, reporting heightened stress levels and reduced HRQOL during periods of limited healthcare access (38). Our findings are consistent with these observations and underscore the importance of contextual factors in interpreting PROs.

### Implications for clinical practice

PROMIS pediatric instruments demonstrated value in capturing dimensions of health that extend beyond traditional clinical markers, offering a more comprehensive perspective on patient wellbeing. This is particularly relevant in pediatric IBD, where symptoms often poorly correlate with inflammation or complications (9, 34). The independence of PROMIS measures from objective markers underscores their potential as complementary tools for monitoring patient health in clinical settings.

Additionally, the alignment of PROMIS trends with patient-reported self-perception of disease further supports their utility. Participants reporting the notion of stable (“feeling the same”) or improved disease (“feeling better”) perception exhibited corresponding improvements in PROMIS scores, particularly in domains such as Global Health, Life Satisfaction, and Pain Interference. PROMIS trends, particularly in domains such as Global Health and Life Satisfaction, appeared to align with patient-reported perceptions of disease stability. However, these findings are exploratory, as no direct statistical associations were tested. These findings highlight the importance of integrating patient perspectives into disease management and clinical decision-making.

### Strengths and limitations

The longitudinal design of this study, combined with standardized disease assessment and repeated measures using LMM, enhances the robustness of our findings. By capturing temporal changes in PROMIS scores over an 18-month follow-up period, this study contributes to the growing body of evidence supporting the use of PROs in pediatric CD.

However, the study has several limitations. The relatively small sample size limits the generalizability of our findings, and the exclusion of recently diagnosed or hospitalized patients reduces the applicability of our results to broader pediatric IBD populations. Furthermore, while PROMIS measures demonstrated trends aligning with clinical improvement, sensitivity analyses or statistical tests testing associations between PROMIS responsiveness and clinical markers were not performed, emphasizing the exploratory nature of these findings and restricting our ability to fully evaluate the responsiveness of these measures. Future studies with larger and more diverse cohorts are needed to address these limitations and validate the clinical utility of PROMIS instruments in pediatric CD.

## Conclusion

This study provides valuable insights into PROMIS instruments' potential clinical utility and responsiveness in pediatric CD care. Over an 18-month follow-up, PROMIS measures captured meaningful changes in health status and HRQOL), demonstrating their value as complementary clinical practice and research tools. However, these findings are exploratory and require further validation in larger, more diverse patient populations.

PROMIS instruments, particularly those measuring Global Health, Pain Interference, and Fatigue, showed significant improvements over time, aligning with trends in clinical and patient-reported outcomes. While these trends suggest that PROMIS measures can capture temporal changes in health and HRQOL, no direct statistical tests of associations between PROMIS and clinical markers were performed. This highlights the need for future research to establish their sensitivity and clinical relevance.

The study underscores the importance of incorporating PROs into disease management. PROMIS instruments provide critical insights into the patient's perspective, offering a holistic understanding of disease impact that complements traditional clinical markers. Integrating these measures can support personalized and effective treatment strategies, ultimately enhancing patient-centered care.

Despite the strengths of this longitudinal design, including the use of standardized assessments and robust statistical modeling, limitations such as the small sample size and lack of sensitivity analysis should be considered. Expanding this research to include larger cohorts and other pediatric IBD subtypes, such as ulcerative colitis, will further clarify PROMIS's role in pediatric IBD care.

In conclusion, PROMIS instruments offer promise as valuable tools for monitoring health status and QOL in pediatric CD patients. Our findings underscore the potential of PROMIS instruments to capture meaningful temporal trends in health status and quality of life. These measures complement traditional clinical tools by capturing unique patient perspectives, contributing to more comprehensive and patient-centered disease management. Their integration into routine practice could enhance clinical decision-making and improve outcomes for pediatric IBD patients.

Further research is warranted to confirm these findings and establish PROMIS to establish their responsiveness to clinical changes and applicability in diverse clinical contexts.

## Data Availability

The original contributions presented in the study are included in the article/Supplementary Material, further inquiries can be directed to the corresponding author.
